# 

**Published:** 1919-01-18

**Authors:** 


### PASSING EVENTS AND TOPICS.

### Dartford's Wooden Town Dismantled.

The dismantling of the American Base Hospital outside Dartford proceeds apace. All but a few of the large buildings are being removed either to other hospitals in England or to France. The patients now number fewer than 100, and these not cot cases. The greater portion of the medical staff and nurses have been transferred to France.

### Demobilise the Blinds.

Ilford's medical officer has a theory about influenza—to which he joins catarrhs and tuberculosis. There is no doubt, he says, that the lack of proper ventilation caused by the lighting restrictions has been an important agent in making us more susceptible to these complaints during the last year or two, and he calls upon residents to remove the blinds and shades which were put up in obedience to D.O.R.A. in the time of air-raids. His suggestion appeals to common sense. Let us by all means have sweetness and light.

### Late Sir C. Wyndham as Surgeon.

Sir Charles Wyndham, the distinguished actor, who died on Sunday in his eighty-second year, was the son of a London practitioner, and was himself trained for the medical profession, becoming in due course, M.R.C.S. London. For some time he served as surgeon in the Federal Army and was present at several of the famous battles in the American Civil War. But the lure of the footlights had a powerful attraction for him early in life, and after some notable appearances as an amateur he succumbed to the ruling passion and forsook surgery for the career of actor. His grace, subtlety, and charm quickly secured for him a foremost position among popular favourites.

### Q.M.N.G. Ceasing Operations.

With the cessation of hostilities the main purpose of the Queen Mary Needlework Guild will have disappeared, and Her Majesty has therefore decided to close the Guild, with the exception of the orthopædic branches, on January 31. Lady Lawley, honorary secretary, in making the communication, speaks of the Queen's "high appreciation of the wonderful spirit" shown on behalf of the sick and wounded, and "pride in the record of work done." A substantial balance in hand is to be devoted to a special scheme, associated with the name of the Guild, for the benefit of disabled sailors and soldiers. Her Majesty extends an invitation to workers who are not already members of their own Guilds to enrol as members of her permanent London Needlework Guild.

### Clever Decorators at St. Mary's.

The sisters and nurses of St. Mary's Hospital were presented with a fine test of ingenuity in providing the usual Christmas decorations. They must have felt as did the Israelites when set to make bricks without straw. The instruction to the staff read: "No gas or electric brackets or pendants to be decorated, nor any flags, bunting, or other draperies used. Nails, tacks, or pins must not be used for the purpose of fixing decorations." And yet the wards of St. Mary's, as recorded in the pages of The Hospital last week, were glorious in their finery when Christmas morning dawned. How did they accomplish the miracle?

### Gold and Silver Tributes.

Collections of gold and silver for the Red Cross have been received by the Committee. The collection made by Lady Sandhurst in Eaton Square profited the fund to the extent of £517. Mr. Sydney Morse collected in Avilie Gardens silver worth £345; Lady Margaret Littleton's effort in Warwick Square realised £103; Lady Stamfordham, Lady Maud Warrender, Lady Mary Hope, and Lady William Neville each contributed a collection worth £100 by activities, respectively, in St. James's Palace, Great Cumberland Place, Lowndes Square, and Onslow Gardens.

### Medals for V.A.D.'s.

Viscount Chilston, better known to the public perhaps as Mr. Akers-Douglas, once a Conservative Whip, says the Government, he understands, intends to give medals to members of the V.A.D. who have worked at home. His lordship, who as County Director for England is in a position to know what is going on behind the scenes in matters affecting the V.A.D., further states that the Minister of Health—when he arrives—may be in a position to find openings for voluntary efforts.

### £60,000 for Glasgow University.

To endow chairs of Bacteriology, Organic Chemistry, and Physiological Chemistry Mr. William Guthrie Gardiner and Mr. F. C. Gardiner, who are prominent in the commercial world of Glasgow, have given the magnificent sum of £60,000 to the University.

### Sheffield Chair of Anatomy.

Miss Alice Jackson, of Sheffield, has left £1,000 to Sheffield University for the "Arthur Jackson" Chair of Anatomy which was founded by the deceased lady's husband.

### Was It the Flea?

A Danish physician, according to the daily Press, is credited with having made the statement that "the miasmatic character of the influenza epidemic shows that it is brought about by infection from fleabites." Since the mosquito was found guilty of being the carrier of tropical diseases, the flea, and his bigger relative the fly, have in more temperate zones been accused of a good many of the scourges which from time to time afflict the human race. Both these pests are wicked enough for anything, and as somebody or something must be blamed for the Spanish influenza it might as well be the flea. All the same, the ladies and gentlemen of high degree who have been "down" with influenza, and not been ashamed of it, will repel with scorn the theory of association with the lively insect.

### London School of Economics.

The time-table for the Lent term of the London School of Economics and Political Science, Portugal Street, W.C., is full of interesting features, the term lasting from January 16 to March 26.

### Artificial Limbs for the West.

At the special request of the Red Cross authorities in London a depôt for the making of provisional artificial limbs is to be opened in Plymouth next month. The premises, 7 The Esplanade, are being fitted out with plant, machinery, and tools for making the limbs. It is hoped the whole of the work will be voluntary. Several ladies are at present receiving instruction in London. Mrs. Grigg, vice-president of the Plymouth division, says the scheme is going to be a very big thing. They would supply provisional limbs for the counties of Devon, Cornwall, and Somerset. The number would run into thousands. "It is," she added, "going to be a wonderful work."

#### Bank Dividend.

The Directors of the London County, Westminster and Parr's Bank, Ltd., have declared a dividend of 10 per cent. for the past half-year (less income tax), making a total distribution of 20 per cent. for the year 1918.

### Forthcoming Events.

Royal College of Surgeons.—Six Hunterian lectures on "Phases in the Life and Work of John Hunter" will be delivered in the theatre of the College in Lincoln's Inn Fields, by Professor Arthur Keith, M.D., LL.D., F.R.S., F.R.C.S., Conservator of the Museum, on January 20, 22, 24, 27, 29, and 31, the time each day being 5 p.m. The lectures will be illustrated by Hunterian preparations, drawings, and records.

Royal Institute of Public Health.—A course of lectures and discussions on "Public Health Problems under War and After-War Conditions" is being held in the Lecture Hall of the Institute, 37 Russell Square, London, W.C. 1. The time is 4 p.m., and the day Wednesday in each week up to March 12. For Wednesday, January 22, the subject is, "Industrial Hygiene in Relation to War Strain and Technical Development," by Professor I. Walker Hall, M.P. The Right Hon. the Lord Moulton of Banff, G.B.E., LL.D., M.A., F.R.S., is announced as chairman.

Combating Venereal Disease.—Mr. E. B. Turner, F.R.C.S., president of the medical committees of the National Council for Combating Venereal Disease, will address a special meeting at the Central Hall, Westminster, on Tuesday, January 28, at 7 p.m., on "Venereal Disease—an urgent Health Problem." Mr. P. Rockliff (President of the Faculty of Insurance) will open a discussion on "Prophylactic Treatment." Sir Kingsley Wood, M.P., L.C.C., is to preside. Tickets may be obtained from the Secretary, Faculty of Insurance, Sicilian House, Southampton Row, W.C. 1.

